# The contribution of postnatal steroid administration to early brain damage in preterm babies with bronchopulmonary dysplasia

**DOI:** 10.3906/sag-2101-295

**Published:** 2021-08-30

**Authors:** Sabahattin ERTUĞRUL, Savaş Mert DARAKCİ, İbrahim KAPLAN, İlyas YOLBAŞ, İbrahim DEGER, Sibel TANRIVERDİ YILMAZ, Şerafettin AKTAŞ

**Affiliations:** 1 Department of Pediatrics, Division of Neonatology, Faculty of Medicine, Dicle University, Diyarbakır Turkey; 2 Department of Pediatrics, Faculty of Medicine, Dicle University, Diyarbakır Turkey; 3 Department of Biochemistry, Faculty of Medicine, Dicle University, Diyarbakır Turkey

**Keywords:** Bronchopulmonary dysplasia, neuron-specific enolase, microtubule-associated proteins, glial fibrillary acidic protein, S100 proteins

## Abstract

**Background/aim:**

Postnatal corticosteroids are commonly used to treat bronchopulmonary dysplasia (BPD). We aimed to show whether S100 calcium-binding B (S100B), neuron-specific enolase (NSE), Tau protein or microtubule-associated protein tau (MAPT), and glial fibrillary acid protein (GFAP) levels would provide any evidence of early neurological damage in premature infants receiving postnatal low dose dexamethasone therapy for BPD treatment.

**Materials and methods:**

In this cohort study, 136 preterm infants diagnosed with BPD at ≤32 weeks of gestation formed the study group, and 64 preterm infants formed the control group. NSE, S100B, GFAP, and MAPT levels were first measured before the postnatal corticosteroid treatment in both the patient and the control group on the 28th day and, for a second time, after treatment termination in the patient group.

**Results:**

There were significant differences between the measured GFAP, MAPT, and NSE values of the BPD and control groups on the 28th day, whereas there was no significant difference between the measured S100B values of the two groups. There were a statistically significant difference between the NSE values measured on the 28th day and after the treatment within the BPD group, whereas no significant difference existed between the GFAP, MAPT, and S100B values.

**Conclusion:**

NSE levels, which indicate brain damage in the early period, increased in preterm babies with BPD who had been administered postnatal dexamethasone.

## 1. Introduction

Bronchopulmonary dysplasia (BPD) is a chronic lung disease that affects more than 40% of preterm newborns [1]. Postnatal corticosteroids (CS) are generally used to prevent or treat BPD in preterm infants [2,3]. Dexamethasone is the most commonly used agent for this purpose [4]. Postnatal CS use, particularly dexamethasone, has been associated with adverse neurodevelopmental outcomes, particularly cerebral palsy [5,6,7]. The DART (dexamethasone: a randomized trial) study showed that low-dose dexamethasone may provide short term advantages without significantly increasing the risk of long-term neurological problems in preterm newborns [8].

Neurobiomarkers (NBs) seem to provide physicians with promising helpful data for the early detection of brain injury in daily clinical practice. Some studies have shown that multiple biomarkers, rather than a single biomarker, may provide additional precision in the early evaluation of cases at risk of neonatal neuronal damage [9]. Therefore, we studied four NBs indicating brain damage, namely S100 calcium-binding B (S100B) proteins, neuron-specific enolase (NSE), Tau protein or microtubule-associated protein tau (MAPT), and glial fibrillary acid protein (GFAP). Although several studies show long term neurodevelopmental outcomes of postnatal CS use for the treatment of preterm infants, no study has yet shown acute neurological injury occurring during that treatment. In this study, we aimed to show whether NSE, S100B, MAPT, and GFAP levels would provide evidence of early neurological damage in premature infants receiving postnatal low-dose dexamethasone therapy to treat BPD.

## 2. Materials and methods

### 2.1. Study design 

This cohort study was carried out between January 2018 and June 2019 in Dicle University Medical Faculty Neonatal Intensive Care Unit. The study protocol was approved by the Dicle University Faculty of Medicine Ethics Committee (21.12.2017-03), and the study was conducted in compliance with the universal principles of the Helsinki Declaration. Written and informed consent was obtained from the parents of all participants. Clinical data and blood samples were collected prospectively. 

### 2.2. Study groups

Group 1 (patient group): patients who were born at ≤32 weeks of gestation, diagnosed with BPD, in need of respiratory support on the postnatal 28th day, and administered low dose dexamethasone treatment.

Group 2 (control group): patients who were born at ≤32 weeks of gestation, and who received no respiratory support on the postnatal 28th day.

### 2.3. Inclusion criteria

1- Preterm babies born at ≤32 (22 0/7-32 6/7) gestational week.

2- Patient group: On the postnatal 28th day, patients who needed respiratory support (in the form of conventional mechanical ventilation, nasal continuous positive airway pressure, or oxygen therapy in the incubator) but had no evidence of infection were diagnosed with BPD.

3- Control group: patients who had no need for respiratory support on the postnatal 28th day.

### 2.4. Exclusion criteria

1- Having chromosomal disorders (such as trisomy 13, 18, 21),

2- Having major congenital malformations,

3- Receiving corticosteroid therapy for any reason between birth and the 28th day of life,

4- Having a history of maternal chorioamnionitis,

5- Having patent ductus arteriosus,

6- Having intraventricular bleeding,

7- Having any proven infection.

### 2.5. Exclusion criteria for discontinuation of glucocorticoid therapy

1- Suspected infection,

2- Nutritional intolerance,

3- Clinical deterioration,

4- Necrotizing enterocolitis,

5- Gastrointestinal bleeding,

6- Intracranial bleeding,

7- Dexamethasone use for extubation.

### 2.6. Dexamethasone therapy

In accordance with the DART study protocol [8], patients with BPD were administered intravenous low-dose dexamethasone therapy at a cumulative dose of 0.89 mg/kg for 10 days.

### 2.7. Collection of samples

In the patient group participated in the study, the first venous blood sample was taken immediately before low-dose dexamethasone treatment was administered on the postnatal 28th day, and then the treatment was started. A second venous blood sample was taken 12 h after the last dose of treatment was administered. Control venous blood sample was taken on the postnatal 28th day in the control group. Blood samples were taken from the median cubital vein in the antecubital fossa. Serum samples were then stored at –80 °C until analysis.

### 2.8. Laboratory analysis

A commercial enzyme-linked immunosorbent assay (ELISA) was used to measure NSE, S100B, GFAP, and MAPT (Shanghai Sunredbio Technology Co., Ltd, Shanghai, China). Biomarkers were measured in ng/mL with the ELISA method. The catalog numbers of NSE, S100B, GFAP, and MAPT ELISA kits used in the study are 210-12-0938, 201-12-4851, 201-12-2095, and 20112-4259, respectively.

As stated below in the manufacturer’s instructions, each of the four ELISA kits were studied separately. The kit uses a double-antibody sandwich ELISA to assay the level of human kit in samples. The kit is added to monoclonal antibody enzyme well, which is pre-coated with human kit monoclonal antibody, incubation, then, (kit) antibodies labeled with biotin and combined with Streptavidin-HRP were added to form immune complex. Then, incubation and washing were carried out again to remove the uncombined enzyme. Later, when chromogen solution A, B was added, the color of the liquid changed into the blue, and, as a result of the effect of the acid, the color finally becomes yellow. The chroma of color and the concentration of the human substance kit of sample were positively correlated.

### 2.9. Statistical analysis

The Statistical Package for Social Science (version 22; IBM Corp. Armonk, NY, USA) was used for statistical analysis. Normality of the distribution of numerical data was examined using Shapiro–Wilk test. Inter-group differences were compared using unpaired Student t-test. Normally distributed numerical variables were presented as mean (SD). Skewed numerical variables were presented as median (interquartile range). Inter-group differences were compared using Mann Whitney–U test. Ordinal variables were compared using χ2 test for trend; comparison between two paired non-parametric variables was performed with Wilcoxon rank test. Correlations were examined with Pearson product-moment correlation for parametric numerical variables, and Spearman rank correlation for non-parametric numerical variables, as appropriate.

Receiver-operating characteristic (ROC) curve analysis was used to examine the value of continuous variables. To assess the overall accuracy of the ROC analyses, the area under the curves (AUCs) were measured. Tests with a p value of less than 0.05 were considered statistically significant.

## 3. Results

In this study, the BPD group consisted of 73 (53.7%) boys and 63 (46.3%) girls, making a total of 136 patients; the control group consisted of 37 (57.8) boys and 27 (42.2%) girls, making a total of 64 preterm infants. There was no statistically significant difference between the groups regarding sex distribution (p = 0.347). In the BPD group, 88.5% (n = 108) of the patients had a history of antenatal steroid use, while the corresponding figure in the control group was 63% (n = 34) (p ≤ 0.001). The mean gestational age (weeks) was 27.62 ± 2.75 in the BPD group and 28.00 ± 1.45 in the control group; the mean birth weight was 1008 ± 307 g in the BPD group and 1068 ± 179 g in the control group (p = 0.299, p = 0.155, respectively).

In the BPD group, no significant difference was found between patients with a history antenatal steroid use and those without with regard to the 28-day GFAP, MAPT, NSE, and S100B values. In addition, no significant difference was found between the BPD and control groups for the same parameters (p = 0.215, p = 0.835, p = 0.618, p = 0.653, respectively).

There was a significant difference between the BPD group and the control group with respect to the GFAP, MAPT, and NSE values measured on the 28th day, whereas no significant difference was found between the S100B values of both groups (Table 1). A significant difference was found between the NSE values but not GFAP, MAPT, and S100B values measured on the 28th day and after the treatment in the BPD group (Table 2).

**Table 1:** Comparison of measured values of GFAP (ng/mL), MAPT (ng/mL), NSE (ng/mL), and S100B (ng/mL) on 28 days of BPD group, and control group cases.

	BPD group (n = 136)	Control group (n = 64)	
Parameters*	Mean ± SD	Mean ± SD	p
GFAP	1.530 ± 4.372	0.91 ± 1.019	0.019
MAPT	24.22 ± 31.01	24.56 ± 27.34	0.012
NSE	8.86 ± 14.48	5.19 ± 5.094	0.000
S100B	361.16 ± 396.40	392.12 ± 389.5	0.465

**Table 2:** Comparison of GFAP (ng/mL), MAPT (ng/mL), NSE (ng/mL), and S100B (ng/mL) values of BPD group on 28th day and after treatment.

	Negative Ranks	Positive Ranks	
Parameters	N (Mean Rank)–(Sum of Ranks)	N (Mean Rank) – (Sum of Ranks)	p*
GFAP	64 (57.63) – (3688)	59 (66.75) – (3938)	0.752
MAPT	136 (55d) – (60,29)	66 (61.59)- (4065)	0.333
NSE	46 (59.11) – (2719)	75 (62.16) – (4662)	0.012
S100B	61 (58.20)v(3550)	62 (65.74)v(4076)	0.507

* Wilcoxon signed ranks test.

There was a significant correlation between the GFAP, MAPT, NSE, and S100B values measured on the 28th day and other parameters in the BPD group (Table 3).

**Table 3:** Correlation between GFAP (ng/mL), MAPT (ng/mL), NSE (ng/mL), and S100B (ng/mL) values and other parameters of the cases measured on the 28th day.

Parameters	GFAP		MAPT		NSE		S100B	
	r	p**	r	p**	r	p**	r	p**
GFAP*			0.484	<0.001	0.435	<0.001	0.618	<0.001
MAPT*	0.484	<0.001			0.538	<0.001	0.568	<0.001
NSE*	0.435	<0.001	0.538	<0.001			0.458	<0.001
S100B*	0.618	<0.001	0.568	<0.001	0.458	<0.001		
								

*28 days measured value, **Spearman’s rho.

ROC analysis results of GFAP, MAPT, NSE and S100B values measured on the 28th day in the BPD group are given in Table 4. See Figure for the ROC curve graph.

**Table 4:** Sensitivity and specificity at cut-off points of GFAP (ng/mL), MAPT (ng/mL), NSE (ng/mL) and S100B (ng/mL) values measured on the 28th day for BPD diagnosis.

Indicators	Area under the ROV curve	(%95 CI)		Cutt-of point(ng/mL)	p	Sensitivity (%)	Specifícity (%)
GFAP	0.600	0.510	0.690	0.588594	0.023	61.5	39.1
MAPT	0.607	0.510	0.704	17.28005	0.015	61.5	37.5
NSE	0.680	0.594	0.766	4.216890	<0.001	65.2	34.4
S100B	0.529	0.430	0.627	234.4936	0.513	50.5	50.0

**Figure:**
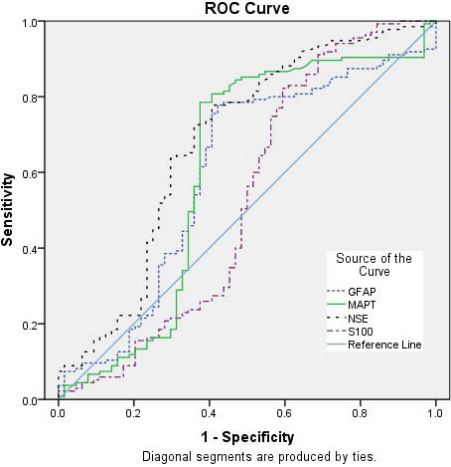
ROC curve graph of GFAP, MAPT, NSE and S100B values.

## 4. Discussion

Detection of perinatal brain damage in the acute phase in term and preterm newborns is still an unsolved problem. Currently, studies to find a reliable biomarker that can predict perinatal brain damage in high-risk newborns are still ongoing [9]. While severe neurodevelopmental deficiencies (17% to 59%) occur in short-term follow-up in premature neonates between 22 and 25 weeks of gestation, it is generally related with a mortality rate as high as 50%. Long-term negative consequences include cerebral palsy (9% to 18%), mental disability (5% to 36%), deafness (2% to 4%), and blindness (0.7% to 9%) [10].

NSE is a glycolytic enzyme, which can be detected in high concentrations in the neuron cell cytoplasm and is released into the extracellular space in cell death [9]. It is an early biochemical predictor of brain damage in newborns and can also help us understand the extent of neuronal damage and assess its prognosis [11]. The S100B protein is highly specific for the nervous system, and is secreted in large amounts from astrocytes into the blood and cerebrospinal fluid (CSF) in neuronal tissue damage [9]. GFAP is an intermediate filament protein of the cytoskeleton in astroglia in the nervous system and is rapidly released into the blood in brain injury [12,13]. MAPT is a microtubule-related structural protein that is released from neurons into CSF or blood when brain damage occurs [14].

Antenatal CS has a proven benefit in preventing complications by ensuring rapid maturation of organs that do not show the desired development in the fetal life. Although these drugs have clinically proven positive effects, they can also trigger some negative developmental side effects [15]. In our study, no statistically significant difference was found between patients with and without a history of antenatal CS use with respect to the brain damage neurobiomarkers including GFAP, MAPT, NSE, and S100B measured on the 28th day (p = 0.215, p = 0.835, p = 0.618, p = 0.653, respectively).

Bronchopulmonary dysplasia is a chronic lung disease that occurs as a result of barotrauma and oxygen toxicity caused by mechanical respiratory support in extremely low birth weight premature neonates. It is a disease with elevated morbidity and mortality risk. CSs play a major role in the treatment of BPD resulting from lung inflammation. CS treatment shows positive effects by rapidly improving lung function, but studies have shown that this treatment may also be associated with several serious side effects in the long term [16]. Systemically administered dexamethasone is the most commonly used CS to prevent and treat BPD. It has alarming long term negative effects associated with its use, the worst of which is cerebral palsy [17]. Doyle et al. [8] found no strong relationship with its use and long-term morbidity although the research failed to provide conclusive evidence about the long term effects of low dose dexamethasone after the first week of life in ventilator-dependent neonates. However, Qin G et al. [5] showed that postnatal dexamethasone use is related with an elevated risk of neurodevelopmental impairment in extremely preterm newborns.

A large number of studies have been conducted to show brain injury in newborn infants, with most being on hypoxic-ischemic encephalopathy (HIE). It was reported that the concentrations of GFAP, S-100, and NSE were significantly elevated in the cerebrospinal fluid of asphyxiated newborns and significantly associated with other markers of long term prognosis and brain injury at 1 years of age [17]. Chaparro-Huerta et al. [18] showed that serum levels of proinflammatory cytokines, S-100B, and NSE were significantly elevated in newborns with brain injury compared to controls. In another study, Lu et al. [19] suggested that in preterm infants, cord blood S100B had the highest sensitivity for brain injury. Several studies have shown changes in serum Tau protein levels in brain injury of the newborn [14]. In our study, a statistically significant difference was found between the GFAP, MAPT, and NSE values measured on the 28th day of the BPD group and the control group, while there was no significant difference between the S100B values. Summanen et al. [20] showed that cord blood S100B in term neonates is a weak predictor of brain injury. In our study, when the correlation between measured NB values and other parameters was evaluated on the 28th day of the cases, GFAP, MAPT, NSE and S100B values showed significant correlations with each other.

Cerebrospinal fluid’sNSE concentrations provide invaluable information as a clinical reflection of the severity of hypoxic-ischemic neuronal damage, and this information may be a harbinger of an abnormal result at two years of age [21]. In a previous study, NSE levels were correlated to neonatal HIE grade and an adverse outcome. The findings suggested that NSE is an accurate biochemical marker for early prediction of hypoxic ischemic brain injury in asphyctic newborns [22]. Sun et al. [23] recently reported that S100B and CSF-NSE levels were correlated to neurodevelopmental outcomes. Catherine et al. [24] evaluated whether serum levels of neuronal biomarkers correlated to neurodevelopmental outcomes in term neonates at 18 months who hypoxic ischemic encephalopathy and therapeutic hypothermia. They showed that serum neuronal biomarkers, S100B, and NSE levels were not correlated to long-term neurodevelopmental outcomes in that population. The predictive ability of serum NSE levels for poor outcomes appears to be better than predictive ability for moderate or severe HIE. The sensitivity and specificity values of serum NSE (cut-off value 40.0 μg/L) as a predictor of moderate to severe HIE were 79% and 70%, respectively, and as a predictor of poor outcomes (cut-off value 45.4 μg/L) it had a sensitivity of 84% and a specificity of 70% [25].

A systematic review showed that NSE may be a sensitive and quantitative NB of neuronal damage [13]. In our study, a significant difference was found between the NSE values measured on the 28th day and after the treatment in the BPD group, while there was no significant difference between the measured GFAP, MAPT, and S100B values. In our study, sensitivity and specificity values for NSE, GFAP, and MAPT were 65.2% and 34.4% (cut-off value 4.21 ng/mL), 61.5% and 39.1% (cut-off value 0.59 ng/mL), and 61.5% and 37.5% (cut-off value 17.28 ng/mL), respectively. The sensitivity and specificity values of S100B were 50.5% and 50.0% (cut-off value 234.49 ng/mL), respectively.

No study has yet shown any evidence of neurological injury in the early period (>28 days) in patients with BPD. In this study, we found that the BPD group had higher levels of neuronal injury NBs than the control group. This finding indicates that respiratory distress experienced by BPD patients may cause neuronal injury in the early period. It is also unknown how much exposure to CS given for treatment contributes to neurological injury early in life. In our study, we aimed to show whether the lowest cumulative dose of postnatal dexamethasone exposure contributes to this brain injury in premature babies diagnosed with BPD. Statistically, we observed that there was a slight increase in NSE levels after treatment discontinuation in the patient group. This finding raises the question that CS can be associated with brain injury, even at low doses. What will be the long term clinical consequences of this increase in premature babies is an issue to be investigated.

This study has some limitations. Firstly, the diagnosis of BPD is made on the 28th day in premature patients who need respiratory support. Secondly, the long-term clinical results of the data obtained in such short term in this study are unknown. However, our study was aimed at obtaining evidence of early brain injury, and it has already planned to follow up these patients and evaluate their future clinical results in the future. Another issue worth mentioning which is the NBs used for demonstrating brain injury, was also studied mostly in CSF in earlier studies but in blood in our study. The reason is that, in our study, an invasive attempt was not made to collect CSF samples from premature newborns due to ethical limitations.

This study showed that the levels of neurobiomarkers indicating early brain injury are increased in preterm babies with BPD. Although our study cannot provide conclusive evidence about the long term effects of low-dose dexamethasone after the first four weeks of life in premature neonates with BPD, it still suggests that there may be a relationship between NSE levels and neurodevelopmental outcome. However, well-designed studies with a greater number of patients using multiple neurobiomarkers are needed.

## Informed consent

The study protocol received institutional review board approval (Dicle University Faculty of Medicine Ethics Committee; date: 21.12.2017/no: 03), and all participants provided signed informed consent form in the format required by the relevant authorities.
